# 2,2′-Bi(9,9-di­ethyl­fluorene)

**DOI:** 10.1107/S1600536814001378

**Published:** 2014-01-22

**Authors:** Ki-Min Park, Hankook Oh, Youngjin Kang

**Affiliations:** a Research Institute of Natural Sciences, Gyeongsang National University, Jinju 660-701, Republic of Korea; b Division of Science Education, Kangwon National University, Chuncheon 200-701, Republic of Korea

## Abstract

The title compound, C_34_H_34_, systematic name 9,9,9′,9′-tetra­ethyl-2,2′-bi(9*H*-fluorene), crystallized with two crystallographically independent mol­ecules (*A* and *B*) in the asymmetric unit. These differ mainly in the orientation of the lateral ethyl chains: in mol­ecule *A*, they are both on the same side of the mol­ecule whereas in mol­ecule *B*, one di­ethyl­fluorene moiety has undergone a 180° rotation such that the two pairs of ethyl residues appear on opposite sides of the mol­ecule. The fluorene ring systems subtend dihedral angles of 31.37 (4) and 43.18 (3)° in mol­ecules *A* and *B*, respectively. Hence the two fluorene moieties are tilted slightly toward one another. This may be due to the presence of inter­molecular C—H⋯π inter­actions between neighboring mol­ecules. The lateral ethyl chains (excluding H atoms) are also almost planar, with each pair almost perpendicular to the plane of the fluorene system to which they are attached with dihedral angles between the ethyl and fluorene planes in the range 86.04 (8)–89.5 (1)°.

## Related literature   

For details of conductive small mol­ecules and their applications in organic electronics, see: Chao *et al.* (2005 ▶); Gong & Lagowski (2008 ▶); Hapiot *et al.* (2005 ▶). For details of the synthesis of the title compound, see: Hapiot *et al.* (2005 ▶). For the crystal structures of other fluorene derivatives, see: Han *et al.* (2006 ▶); Jasinski *et al.* (2003 ▶); Suchod *et al.* (2000 ▶).
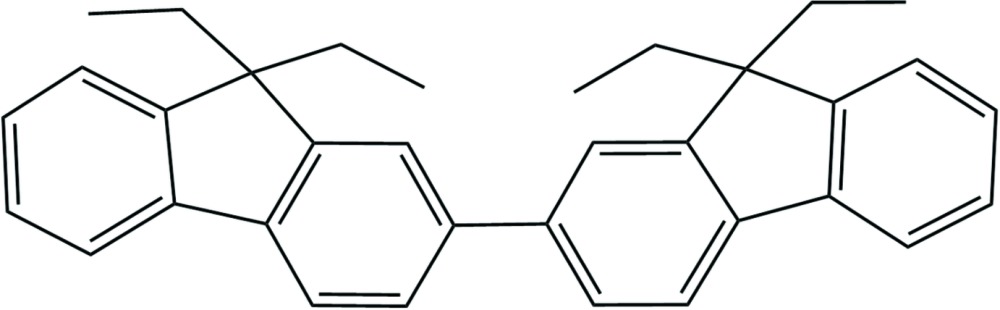


## Experimental   

### 

#### Crystal data  

C_34_H_34_
*M* *_r_* = 442.61
Triclinic, 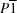

*a* = 12.3149 (6) Å
*b* = 14.8415 (7) Å
*c* = 15.8795 (8) Å
α = 69.725 (1)°
β = 89.368 (1)°
γ = 73.433 (1)°
*V* = 2597.0 (2) Å^3^
*Z* = 4
Mo *K*α radiation
μ = 0.06 mm^−1^
*T* = 173 K
0.40 × 0.35 × 0.25 mm

#### Data collection  

Bruker APEXII CCD area detector diffractometer
Absorption correction: multi-scan (*SADABS*; Sheldrick, 1996 ▶) *T*_min_ = 0.975, *T*_max_ = 0.984
14768 measured reflections
10001 independent reflections
7320 reflections with *I* > 2σ(*I*)
*R*_int_ = 0.032

#### Refinement  

*R*[*F* ^2^ > 2σ(*F* ^2^)] = 0.059
*wR*(*F* ^2^) = 0.135
*S* = 1.08
10001 reflections
613 parameters
H-atom parameters constrained
Δρ_max_ = 0.27 e Å^−3^
Δρ_min_ = −0.26 e Å^−3^

### 

Data collection: *APEX2* (Bruker, 2006 ▶); cell refinement: *SAINT* (Bruker, 2006 ▶); data reduction: *SAINT* (Bruker, 2006 ▶); program(s) used to solve structure: *SHELXS97* (Sheldrick, 2008 ▶); program(s) used to refine structure: *SHELXL97* (Sheldrick, 2008 ▶); molecular graphics: *SHELXTL* (Sheldrick, 2008 ▶); software used to prepare material for publication: *SHELXTL*.

## Supplementary Material

Crystal structure: contains datablock(s) I, global. DOI: 10.1107/S1600536814001378/sj5384sup1.cif

Structure factors: contains datablock(s) I. DOI: 10.1107/S1600536814001378/sj5384Isup2.hkl

Supporting information file. DOI: 10.1107/S1600536814001378/sj5384Isup3.cml

CCDC reference:

Additional supporting information:  crystallographic information; 3D view; checkCIF report

## Figures and Tables

**Table 1:** Hydrogen-bond geometry (Å, °) *Cg*3 and *Cg*4 are the centroids of the C14–C16/C24–C26 and C8–C13 rings, respectively.

*D*—H⋯*A*	*D*—H	H⋯*A*	*D*⋯*A*	*D*—H⋯*A*
C43—H43⋯*Cg*3^i^	0.95	2.64	3.49	150
C60—H60⋯*Cg*4^i^	0.95	3.15	3.83	130

Symmetry code: (i) 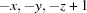.

**Fig. 1.**
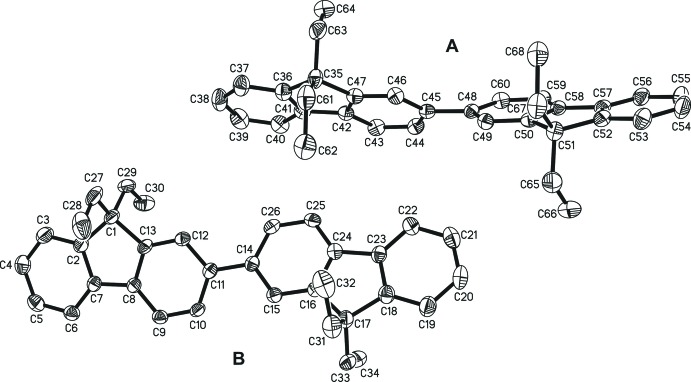
The asymmetric unit of the title compound. Displacement ellipsoids are drawn at the 50% probability level. Hydrogen atoms are omitted for clarity.

## supplementary crystallographic information

### 1. Comment

As a potential conductive small molecule, 2,2'-bi(9,9-diethylfluorene) is regarded as one of the most promising candidate materials for organic electronics due to its unique photophysical properties, good thermal stability as well as a stable glass phase at room temperature (Chao *et al.*, 2005; Hapiot *et al.*, 2005). Therefore, the crystal structure of 2,2'-bi(9,9-diethylfluorene) plays key role in understanding the reasons that this compound has a high thermal stability and a stable glass form (Gong & Lagowski, 2008).

The title compound (Scheme 1, Fig.1) crystallized with two crystallographically independent molecules (*A* and *B*) in the asymmetric unit, which differ mainly in the orientation of the lateral ethyl chains in each molecule. The ethyl substituents in *A* are found on the same side of the molecule whereas in molecule B one diethylfluorene moiety has undergone a 180° rotation such that the two pairs of ethyl residues appear on opposite sides of the molecule. In both molecules the fluorene segments are planar with a maximum r.m.s. deviation of 0.048 ° for the C48–C60 ring system. The ethyl chains (excluding H atoms) are also planar, with each pair almost perpendicular to the plane of the fluorene system to which they are attached, with dihedral angles between the ethyl and fluorene planes in the range 86.04 (8)° to 89.5 (1)°. This interruption of π-conjugation of two fluorene segments may be caused by intermolecular intermolecular C—H···π interactions between neighboring molecules (Table 1). All bond lengths and bond angles are normal and comparable to those of observed in the structures of other fluorene derivatives (Han *et al.*, 2006; Jasinski *et al.*, 2003; Suchod *et al.*, 2000).

### 2. Experimental

The title compound was synthesized by a literature method (Hapiot *et al.*, 2005). Slow evaporation of a solution of the title compound in dichloromethane and hexane (1:2 *v*/*v*) gave single crystals suitable for X-ray analysis.

### 3. Refinement

All H-atoms were positioned geometrically and refined using a riding model with d(C—H) = 0.95 Å, *U*_iso_ = 1.2*U*_eq_(C) for aromatic, d(C—H) = 0.99 Å, *U*_iso_ = 1.2*U*_eq_(C) for methylene, and d(C—H) = 0.98 Å, *U*_iso_ = 1.2*U*_eq_(C) for methyl protons.

### Crystal data

**Table:**

C_34_H_34_	*Z* = 4
*M**_r_* = 442.61	*F*(000) = 952
Triclinic, *P*1	*D*_x_ = 1.132 Mg m^−^^3^
Hall symbol: -P 1	Mo *K*α radiation, λ = 0.71073 Å
*a* = 12.3149 (6) Å	Cell parameters from 6198 reflections
*b* = 14.8415 (7) Å	θ = 2.3–28.3°
*c* = 15.8795 (8) Å	µ = 0.06 mm^−^^1^
α = 69.725 (1)°	*T* = 173 K
β = 89.368 (1)°	Block, colourless
γ = 73.433 (1)°	0.40 × 0.35 × 0.25 mm
*V* = 2597.0 (2) Å^3^	

### Data collection

**Table:**

Bruker APEXII CCD area detector diffractometer	10001 independent reflections
Radiation source: fine-focus sealed tube	7320 reflections with *I* > 2σ(*I*)
Graphite monochromator	*R*_int_ = 0.032
φ and ω scans	θ_max_ = 26.0°, θ_min_ = 1.4°
Absorption correction: multi-scan (*SADABS*; Sheldrick, 1996)	*h* = −12→15
*T*_min_ = 0.975, *T*_max_ = 0.984	*k* = −18→18
14768 measured reflections	*l* = −19→14

### Refinement

**Table:**

Refinement on *F*^2^	Primary atom site location: structure-invariant direct methods
Least-squares matrix: full	Secondary atom site location: difference Fourier map
*R*[*F*^2^ > 2σ(*F*^2^)] = 0.059	Hydrogen site location: inferred from neighbouring sites
*wR*(*F*^2^) = 0.135	H-atom parameters constrained
*S* = 1.08	*w* = 1/[σ^2^(*F*_o_^2^) + (0.0373*P*)^2^ + 1.6362*P*] where *P* = (*F*_o_^2^ + 2*F*_c_^2^)/3
10001 reflections	(Δ/σ)_max_ < 0.001
613 parameters	Δρ_max_ = 0.27 e Å^−^^3^
0 restraints	Δρ_min_ = −0.26 e Å^−^^3^

### Special details

**Table:**

Geometry. All e.s.d.'s (except the e.s.d. in the dihedral angle between two l.s. planes) are estimated using the full covariance matrix. The cell e.s.d.'s are taken into account individually in the estimation of e.s.d.'s in distances, angles and torsion angles; correlations between e.s.d.'s in cell parameters are only used when they are defined by crystal symmetry. An approximate (isotropic) treatment of cell e.s.d.'s is used for estimating e.s.d.'s involving l.s. planes.
Refinement. Refinement of *F*^2^ against ALL reflections. The weighted *R*-factor *wR* and goodness of fit *S* are based on *F*^2^, conventional *R*-factors *R* are based on *F*, with *F* set to zero for negative *F*^2^. The threshold expression of *F*^2^ > σ(*F*^2^) is used only for calculating *R*-factors(gt) *etc*. and is not relevant to the choice of reflections for refinement. *R*-factors based on *F*^2^ are statistically about twice as large as those based on *F*, and *R*-factors based on ALL data will be even larger.

### Fractional atomic coordinates and isotropic or equivalent isotropic displacement parameters (Å^2^)

**Table:**

	*x*	*y*	*z*	*U*_iso_*/*U*_eq_	
C1	0.34960 (19)	0.12638 (16)	0.07528 (15)	0.0320 (5)	
C2	0.33666 (18)	0.21742 (16)	−0.01130 (15)	0.0315 (5)	
C3	0.3600 (2)	0.21887 (18)	−0.09731 (16)	0.0408 (6)	
H3	0.3901	0.1578	−0.1081	0.049*	
C4	0.3385 (2)	0.31178 (19)	−0.16778 (16)	0.0425 (6)	
H4	0.3540	0.3138	−0.2270	0.051*	
C5	0.2948 (2)	0.40117 (17)	−0.15222 (16)	0.0366 (5)	
H5	0.2801	0.4637	−0.2010	0.044*	
C6	0.27234 (18)	0.39989 (16)	−0.06604 (15)	0.0303 (5)	
H6	0.2427	0.4610	−0.0553	0.036*	
C7	0.29396 (16)	0.30763 (16)	0.00414 (14)	0.0266 (5)	
C8	0.27622 (16)	0.28309 (15)	0.10035 (14)	0.0254 (4)	
C9	0.23575 (17)	0.34442 (15)	0.14993 (14)	0.0273 (5)	
H9	0.2155	0.4156	0.1219	0.033*	
C10	0.22538 (17)	0.30043 (15)	0.24073 (14)	0.0271 (5)	
H10	0.1978	0.3424	0.2747	0.033*	
C11	0.25446 (16)	0.19547 (15)	0.28429 (14)	0.0247 (4)	
C12	0.29613 (17)	0.13462 (15)	0.23337 (14)	0.0273 (5)	
H12	0.3170	0.0634	0.2612	0.033*	
C13	0.30680 (17)	0.17834 (15)	0.14255 (14)	0.0267 (5)	
C14	0.23690 (16)	0.15167 (15)	0.38076 (14)	0.0245 (4)	
C15	0.24091 (16)	0.20295 (15)	0.43988 (14)	0.0258 (5)	
H15	0.2596	0.2644	0.4188	0.031*	
C16	0.21796 (17)	0.16465 (15)	0.52783 (14)	0.0260 (5)	
C17	0.22179 (18)	0.20635 (17)	0.60234 (15)	0.0310 (5)	
C18	0.17970 (18)	0.13300 (17)	0.67919 (15)	0.0320 (5)	
C19	0.1576 (2)	0.13455 (19)	0.76502 (16)	0.0400 (6)	
H19	0.1666	0.1875	0.7822	0.048*	
C20	0.1221 (2)	0.0573 (2)	0.82521 (17)	0.0438 (6)	
H20	0.1059	0.0581	0.8836	0.053*	
C21	0.1101 (2)	−0.02093 (19)	0.80109 (17)	0.0419 (6)	
H21	0.0866	−0.0734	0.8433	0.050*	
C22	0.13196 (19)	−0.02323 (17)	0.71561 (15)	0.0350 (5)	
H22	0.1238	−0.0768	0.6990	0.042*	
C23	0.16601 (17)	0.05458 (16)	0.65500 (14)	0.0283 (5)	
C24	0.19018 (16)	0.07423 (15)	0.56038 (14)	0.0258 (5)	
C25	0.18838 (18)	0.02103 (15)	0.50411 (15)	0.0293 (5)	
H25	0.1714	−0.0411	0.5260	0.035*	
C26	0.21181 (17)	0.05987 (16)	0.41523 (15)	0.0289 (5)	
H26	0.2108	0.0233	0.3767	0.035*	
C27	0.4756 (2)	0.06177 (19)	0.10259 (17)	0.0448 (7)	
H27A	0.4794	0.0018	0.1567	0.054*	
H27B	0.5042	0.0378	0.0532	0.054*	
C28	0.5540 (2)	0.1161 (2)	0.1227 (2)	0.0598 (8)	
H28A	0.6317	0.0701	0.1395	0.072*	
H28B	0.5279	0.1386	0.1727	0.072*	
H28C	0.5528	0.1745	0.0690	0.072*	
C29	0.2755 (2)	0.06115 (17)	0.06740 (17)	0.0404 (6)	
H29A	0.3030	0.0325	0.0206	0.048*	
H29B	0.2858	0.0043	0.1255	0.048*	
C30	0.1493 (2)	0.11732 (19)	0.04379 (19)	0.0481 (7)	
H30A	0.1081	0.0709	0.0403	0.058*	
H30B	0.1377	0.1725	−0.0146	0.058*	
H30C	0.1206	0.1448	0.0904	0.058*	
C31	0.3473 (2)	0.1978 (2)	0.62692 (17)	0.0421 (6)	
H31A	0.3711	0.2480	0.5769	0.051*	
H31B	0.3503	0.2157	0.6812	0.051*	
C32	0.4327 (2)	0.0948 (2)	0.64500 (18)	0.0504 (7)	
H32A	0.5088	0.0964	0.6602	0.061*	
H32B	0.4328	0.0770	0.5910	0.061*	
H32C	0.4114	0.0445	0.6955	0.061*	
C33	0.1486 (2)	0.31692 (17)	0.57677 (16)	0.0378 (6)	
H33A	0.1510	0.3369	0.6299	0.045*	
H33B	0.1830	0.3596	0.5284	0.045*	
C34	0.0246 (2)	0.33856 (18)	0.54482 (18)	0.0443 (6)	
H34A	−0.0153	0.4103	0.5299	0.053*	
H34B	−0.0114	0.2985	0.5928	0.053*	
H34C	0.0207	0.3208	0.4912	0.053*	
C35	0.38207 (18)	−0.33106 (17)	0.59294 (15)	0.0321 (5)	
C36	0.33463 (18)	−0.27599 (17)	0.49421 (15)	0.0311 (5)	
C37	0.3905 (2)	−0.27170 (19)	0.41756 (16)	0.0387 (6)	
H37	0.4694	−0.3063	0.4225	0.046*	
C38	0.3300 (2)	−0.2161 (2)	0.33283 (17)	0.0444 (6)	
H38	0.3682	−0.2120	0.2799	0.053*	
C39	0.2141 (2)	−0.16648 (19)	0.32548 (17)	0.0433 (6)	
H39	0.1736	−0.1289	0.2675	0.052*	
C40	0.1573 (2)	−0.17142 (17)	0.40182 (16)	0.0360 (5)	
H40	0.0782	−0.1375	0.3966	0.043*	
C41	0.21772 (18)	−0.22685 (16)	0.48666 (15)	0.0292 (5)	
C42	0.18096 (17)	−0.24496 (15)	0.57759 (14)	0.0262 (5)	
C43	0.07414 (17)	−0.21580 (16)	0.60712 (15)	0.0295 (5)	
H43	0.0090	−0.1782	0.5649	0.035*	
C44	0.06406 (17)	−0.24229 (16)	0.69869 (15)	0.0295 (5)	
H44	−0.0090	−0.2232	0.7187	0.035*	
C45	0.15895 (17)	−0.29669 (15)	0.76306 (14)	0.0257 (5)	
C46	0.26606 (17)	−0.32621 (15)	0.73185 (14)	0.0271 (5)	
H46	0.3316	−0.3629	0.7739	0.033*	
C47	0.27660 (17)	−0.30221 (15)	0.64076 (14)	0.0271 (5)	
C48	0.14490 (16)	−0.32232 (15)	0.86046 (14)	0.0247 (4)	
C49	0.22403 (16)	−0.31726 (15)	0.92071 (14)	0.0261 (5)	
H49	0.2878	−0.2954	0.8989	0.031*	
C50	0.20895 (16)	−0.34417 (15)	1.01157 (14)	0.0252 (5)	
C51	0.28110 (18)	−0.34259 (17)	1.08828 (15)	0.0299 (5)	
C52	0.22231 (18)	−0.38944 (16)	1.17006 (15)	0.0295 (5)	
C53	0.2525 (2)	−0.41327 (18)	1.26060 (16)	0.0369 (5)	
H53	0.3198	−0.4034	1.2793	0.044*	
C54	0.1828 (2)	−0.45193 (19)	1.32392 (17)	0.0437 (6)	
H54	0.2034	−0.4694	1.3863	0.052*	
C55	0.0833 (2)	−0.46527 (19)	1.29676 (16)	0.0412 (6)	
H55	0.0361	−0.4909	1.3407	0.049*	
C56	0.05245 (19)	−0.44147 (17)	1.20620 (16)	0.0343 (5)	
H56	−0.0156	−0.4505	1.1877	0.041*	
C57	0.12285 (17)	−0.40417 (15)	1.14287 (15)	0.0278 (5)	
C58	0.11462 (16)	−0.37654 (15)	1.04451 (14)	0.0251 (4)	
C59	0.03473 (17)	−0.38021 (16)	0.98548 (15)	0.0278 (5)	
H59	−0.0297	−0.4009	1.0072	0.033*	
C60	0.05033 (17)	−0.35335 (16)	0.89466 (15)	0.0285 (5)	
H60	−0.0042	−0.3559	0.8544	0.034*	
C61	0.48020 (18)	−0.2970 (2)	0.61827 (17)	0.0404 (6)	
H61A	0.5076	−0.3364	0.6826	0.048*	
H61B	0.5439	−0.3135	0.5823	0.048*	
C62	0.4501 (2)	−0.1860 (2)	0.60386 (19)	0.0496 (7)	
H62A	0.5170	−0.1714	0.6221	0.060*	
H62B	0.3883	−0.1687	0.6402	0.060*	
H62C	0.4257	−0.1460	0.5399	0.060*	
C63	0.4234 (2)	−0.44648 (18)	0.61561 (18)	0.0442 (6)	
H63A	0.4920	−0.4629	0.5841	0.053*	
H63B	0.4463	−0.4800	0.6812	0.053*	
C64	0.3363 (2)	−0.4901 (2)	0.59015 (19)	0.0530 (7)	
H64A	0.3694	−0.5630	0.6069	0.064*	
H64B	0.3147	−0.4592	0.5250	0.064*	
H64C	0.2687	−0.4761	0.6222	0.064*	
C65	0.2708 (2)	−0.23223 (19)	1.07764 (17)	0.0425 (6)	
H65A	0.3184	−0.2335	1.1281	0.051*	
H65B	0.3022	−0.2010	1.0210	0.051*	
C66	0.1507 (2)	−0.16619 (19)	1.07594 (19)	0.0508 (7)	
H66A	0.1523	−0.0983	1.0687	0.061*	
H66B	0.1194	−0.1948	1.1327	0.061*	
H66C	0.1029	−0.1629	1.0254	0.061*	
C67	0.40812 (18)	−0.40071 (19)	1.09331 (16)	0.0387 (6)	
H67A	0.4392	−0.3662	1.0377	0.046*	
H67B	0.4493	−0.3974	1.1448	0.046*	
C68	0.43250 (19)	−0.51055 (19)	1.10397 (17)	0.0448 (6)	
H68A	0.5147	−0.5411	1.1061	0.054*	
H68B	0.3937	−0.5150	1.0527	0.054*	
H68C	0.4048	−0.5464	1.1601	0.054*	

### Atomic displacement parameters (Å^2^)

**Table:**

	*U*^11^	*U*^22^	*U*^33^	*U*^12^	*U*^13^	*U*^23^
C1	0.0391 (13)	0.0252 (11)	0.0252 (12)	0.0006 (9)	0.0053 (9)	−0.0095 (9)
C2	0.0311 (12)	0.0310 (12)	0.0290 (12)	−0.0045 (9)	0.0046 (9)	−0.0104 (10)
C3	0.0546 (15)	0.0333 (13)	0.0322 (14)	−0.0066 (11)	0.0085 (11)	−0.0144 (11)
C4	0.0557 (16)	0.0471 (15)	0.0256 (13)	−0.0171 (12)	0.0088 (11)	−0.0129 (11)
C5	0.0416 (13)	0.0330 (13)	0.0312 (13)	−0.0140 (10)	0.0007 (10)	−0.0044 (10)
C6	0.0297 (11)	0.0261 (11)	0.0327 (13)	−0.0073 (9)	0.0021 (9)	−0.0085 (10)
C7	0.0207 (10)	0.0305 (11)	0.0286 (12)	−0.0065 (8)	0.0029 (8)	−0.0114 (9)
C8	0.0192 (10)	0.0266 (11)	0.0297 (12)	−0.0051 (8)	0.0033 (8)	−0.0107 (9)
C9	0.0251 (11)	0.0228 (11)	0.0331 (13)	−0.0056 (8)	0.0034 (9)	−0.0100 (9)
C10	0.0262 (11)	0.0266 (11)	0.0314 (12)	−0.0060 (9)	0.0062 (9)	−0.0155 (10)
C11	0.0169 (10)	0.0290 (11)	0.0282 (12)	−0.0048 (8)	0.0012 (8)	−0.0118 (9)
C12	0.0261 (11)	0.0210 (10)	0.0299 (12)	−0.0013 (8)	0.0038 (9)	−0.0078 (9)
C13	0.0217 (10)	0.0262 (11)	0.0297 (12)	−0.0007 (8)	0.0033 (8)	−0.0124 (9)
C14	0.0171 (10)	0.0270 (11)	0.0279 (12)	−0.0029 (8)	0.0017 (8)	−0.0110 (9)
C15	0.0227 (10)	0.0259 (11)	0.0299 (12)	−0.0088 (8)	0.0039 (8)	−0.0104 (9)
C16	0.0221 (10)	0.0301 (11)	0.0291 (12)	−0.0095 (9)	0.0029 (8)	−0.0132 (9)
C17	0.0341 (12)	0.0375 (13)	0.0292 (12)	−0.0179 (10)	0.0065 (9)	−0.0156 (10)
C18	0.0287 (11)	0.0387 (13)	0.0308 (13)	−0.0123 (10)	0.0044 (9)	−0.0135 (10)
C19	0.0458 (14)	0.0492 (15)	0.0324 (14)	−0.0195 (12)	0.0095 (11)	−0.0197 (12)
C20	0.0431 (14)	0.0605 (17)	0.0282 (13)	−0.0156 (12)	0.0116 (11)	−0.0165 (12)
C21	0.0393 (14)	0.0458 (15)	0.0354 (14)	−0.0159 (11)	0.0092 (11)	−0.0058 (12)
C22	0.0379 (13)	0.0333 (13)	0.0319 (13)	−0.0123 (10)	0.0049 (10)	−0.0080 (10)
C23	0.0239 (11)	0.0317 (12)	0.0273 (12)	−0.0058 (9)	0.0027 (9)	−0.0103 (10)
C24	0.0206 (10)	0.0286 (11)	0.0258 (12)	−0.0055 (8)	−0.0005 (8)	−0.0082 (9)
C25	0.0329 (12)	0.0217 (11)	0.0316 (13)	−0.0070 (9)	0.0017 (9)	−0.0085 (9)
C26	0.0289 (11)	0.0277 (11)	0.0306 (13)	−0.0037 (9)	0.0028 (9)	−0.0151 (10)
C27	0.0484 (15)	0.0391 (14)	0.0310 (14)	0.0101 (11)	0.0091 (11)	−0.0123 (11)
C28	0.0306 (14)	0.0637 (19)	0.0582 (19)	0.0000 (13)	0.0118 (13)	−0.0008 (15)
C29	0.0617 (16)	0.0244 (12)	0.0347 (14)	−0.0080 (11)	0.0088 (12)	−0.0141 (10)
C30	0.0561 (17)	0.0387 (14)	0.0562 (18)	−0.0176 (12)	0.0030 (13)	−0.0221 (13)
C31	0.0426 (14)	0.0608 (17)	0.0345 (14)	−0.0299 (13)	0.0037 (11)	−0.0192 (12)
C32	0.0372 (14)	0.0680 (19)	0.0410 (16)	−0.0232 (13)	−0.0058 (11)	−0.0072 (14)
C33	0.0509 (15)	0.0391 (13)	0.0378 (14)	−0.0238 (11)	0.0157 (11)	−0.0231 (11)
C34	0.0472 (15)	0.0348 (13)	0.0540 (17)	−0.0097 (11)	0.0133 (12)	−0.0220 (12)
C35	0.0241 (11)	0.0402 (13)	0.0294 (12)	−0.0064 (9)	0.0059 (9)	−0.0120 (10)
C36	0.0303 (11)	0.0348 (12)	0.0322 (13)	−0.0146 (10)	0.0039 (9)	−0.0128 (10)
C37	0.0361 (13)	0.0513 (15)	0.0373 (14)	−0.0200 (11)	0.0100 (10)	−0.0207 (12)
C38	0.0531 (16)	0.0619 (17)	0.0328 (14)	−0.0324 (14)	0.0138 (12)	−0.0227 (13)
C39	0.0518 (16)	0.0513 (16)	0.0314 (14)	−0.0256 (13)	−0.0020 (11)	−0.0121 (12)
C40	0.0364 (13)	0.0382 (13)	0.0348 (14)	−0.0144 (10)	−0.0030 (10)	−0.0120 (11)
C41	0.0276 (11)	0.0304 (12)	0.0324 (13)	−0.0127 (9)	0.0008 (9)	−0.0114 (10)
C42	0.0252 (11)	0.0240 (10)	0.0288 (12)	−0.0075 (8)	0.0008 (9)	−0.0087 (9)
C43	0.0211 (10)	0.0330 (12)	0.0330 (13)	−0.0055 (9)	−0.0033 (9)	−0.0121 (10)
C44	0.0197 (10)	0.0332 (12)	0.0358 (13)	−0.0057 (9)	0.0034 (9)	−0.0144 (10)
C45	0.0235 (10)	0.0244 (11)	0.0299 (12)	−0.0080 (8)	0.0016 (9)	−0.0101 (9)
C46	0.0198 (10)	0.0289 (11)	0.0288 (12)	−0.0049 (8)	−0.0019 (8)	−0.0076 (9)
C47	0.0223 (10)	0.0274 (11)	0.0313 (13)	−0.0072 (8)	0.0018 (9)	−0.0104 (9)
C48	0.0203 (10)	0.0233 (10)	0.0285 (12)	−0.0029 (8)	0.0029 (8)	−0.0097 (9)
C49	0.0198 (10)	0.0274 (11)	0.0310 (12)	−0.0083 (8)	0.0040 (8)	−0.0096 (9)
C50	0.0205 (10)	0.0229 (10)	0.0320 (12)	−0.0049 (8)	0.0017 (8)	−0.0110 (9)
C51	0.0265 (11)	0.0371 (12)	0.0297 (12)	−0.0123 (9)	0.0034 (9)	−0.0141 (10)
C52	0.0277 (11)	0.0290 (11)	0.0319 (13)	−0.0052 (9)	0.0029 (9)	−0.0134 (10)
C53	0.0341 (12)	0.0464 (14)	0.0334 (14)	−0.0108 (11)	0.0014 (10)	−0.0191 (11)
C54	0.0522 (16)	0.0508 (16)	0.0275 (13)	−0.0099 (12)	0.0097 (11)	−0.0179 (12)
C55	0.0468 (15)	0.0462 (15)	0.0339 (14)	−0.0154 (12)	0.0197 (11)	−0.0177 (12)
C56	0.0331 (12)	0.0361 (13)	0.0387 (14)	−0.0117 (10)	0.0130 (10)	−0.0186 (11)
C57	0.0268 (11)	0.0232 (11)	0.0333 (12)	−0.0038 (8)	0.0056 (9)	−0.0128 (9)
C58	0.0230 (10)	0.0227 (10)	0.0298 (12)	−0.0051 (8)	0.0061 (8)	−0.0110 (9)
C59	0.0206 (10)	0.0301 (11)	0.0351 (13)	−0.0095 (9)	0.0072 (9)	−0.0131 (10)
C60	0.0203 (10)	0.0311 (12)	0.0352 (13)	−0.0078 (9)	−0.0017 (9)	−0.0131 (10)
C61	0.0207 (11)	0.0612 (17)	0.0352 (14)	−0.0096 (11)	0.0024 (9)	−0.0146 (12)
C62	0.0348 (14)	0.0692 (19)	0.0551 (18)	−0.0259 (13)	0.0044 (12)	−0.0263 (15)
C63	0.0393 (14)	0.0423 (14)	0.0397 (15)	0.0019 (11)	0.0110 (11)	−0.0125 (12)
C64	0.0702 (19)	0.0364 (14)	0.0520 (18)	−0.0134 (13)	0.0148 (14)	−0.0177 (13)
C65	0.0518 (15)	0.0493 (15)	0.0400 (15)	−0.0296 (13)	0.0086 (12)	−0.0208 (12)
C66	0.0671 (18)	0.0323 (14)	0.0549 (18)	−0.0128 (13)	0.0071 (14)	−0.0195 (13)
C67	0.0236 (11)	0.0597 (16)	0.0342 (14)	−0.0142 (11)	0.0005 (9)	−0.0171 (12)
C68	0.0249 (12)	0.0565 (16)	0.0389 (15)	0.0026 (11)	0.0001 (10)	−0.0120 (12)

### Geometric parameters (Å, º)

**Table:**

C1—C2	1.528 (3)	C35—C36	1.525 (3)
C1—C13	1.530 (3)	C35—C47	1.528 (3)
C1—C29	1.541 (3)	C35—C61	1.542 (3)
C1—C27	1.547 (3)	C35—C63	1.550 (3)
C2—C3	1.387 (3)	C36—C37	1.383 (3)
C2—C7	1.397 (3)	C36—C41	1.401 (3)
C3—C4	1.397 (3)	C37—C38	1.396 (3)
C3—H3	0.9500	C37—H37	0.9500
C4—C5	1.388 (3)	C38—C39	1.392 (4)
C4—H4	0.9500	C38—H38	0.9500
C5—C6	1.388 (3)	C39—C40	1.383 (3)
C5—H5	0.9500	C39—H39	0.9500
C6—C7	1.389 (3)	C40—C41	1.396 (3)
C6—H6	0.9500	C40—H40	0.9500
C7—C8	1.472 (3)	C41—C42	1.466 (3)
C8—C9	1.388 (3)	C42—C43	1.393 (3)
C8—C13	1.399 (3)	C42—C47	1.407 (3)
C9—C10	1.383 (3)	C43—C44	1.383 (3)
C9—H9	0.9500	C43—H43	0.9500
C10—C11	1.405 (3)	C44—C45	1.407 (3)
C10—H10	0.9500	C44—H44	0.9500
C11—C12	1.402 (3)	C45—C46	1.409 (3)
C11—C14	1.483 (3)	C45—C48	1.480 (3)
C12—C13	1.384 (3)	C46—C47	1.379 (3)
C12—H12	0.9500	C46—H46	0.9500
C14—C26	1.404 (3)	C48—C60	1.408 (3)
C14—C15	1.407 (3)	C48—C49	1.408 (3)
C15—C16	1.373 (3)	C49—C50	1.383 (3)
C15—H15	0.9500	C49—H49	0.9500
C16—C24	1.401 (3)	C50—C58	1.409 (3)
C16—C17	1.520 (3)	C50—C51	1.526 (3)
C17—C18	1.526 (3)	C51—C52	1.528 (3)
C17—C33	1.541 (3)	C51—C67	1.544 (3)
C17—C31	1.558 (3)	C51—C65	1.555 (3)
C18—C19	1.394 (3)	C52—C53	1.384 (3)
C18—C23	1.398 (3)	C52—C57	1.401 (3)
C19—C20	1.392 (3)	C53—C54	1.392 (3)
C19—H19	0.9500	C53—H53	0.9500
C20—C21	1.387 (4)	C54—C55	1.391 (4)
C20—H20	0.9500	C54—H54	0.9500
C21—C22	1.391 (3)	C55—C56	1.386 (3)
C21—H21	0.9500	C55—H55	0.9500
C22—C23	1.390 (3)	C56—C57	1.392 (3)
C22—H22	0.9500	C56—H56	0.9500
C23—C24	1.472 (3)	C57—C58	1.468 (3)
C24—C25	1.386 (3)	C58—C59	1.391 (3)
C25—C26	1.389 (3)	C59—C60	1.384 (3)
C25—H25	0.9500	C59—H59	0.9500
C26—H26	0.9500	C60—H60	0.9500
C27—C28	1.521 (4)	C61—C62	1.516 (4)
C27—H27A	0.9900	C61—H61A	0.9900
C27—H27B	0.9900	C61—H61B	0.9900
C28—H28A	0.9800	C62—H62A	0.9800
C28—H28B	0.9800	C62—H62B	0.9800
C28—H28C	0.9800	C62—H62C	0.9800
C29—C30	1.520 (3)	C63—C64	1.524 (4)
C29—H29A	0.9900	C63—H63A	0.9900
C29—H29B	0.9900	C63—H63B	0.9900
C30—H30A	0.9800	C64—H64A	0.9800
C30—H30B	0.9800	C64—H64B	0.9800
C30—H30C	0.9800	C64—H64C	0.9800
C31—C32	1.521 (4)	C65—C66	1.518 (4)
C31—H31A	0.9900	C65—H65A	0.9900
C31—H31B	0.9900	C65—H65B	0.9900
C32—H32A	0.9800	C66—H66A	0.9800
C32—H32B	0.9800	C66—H66B	0.9800
C32—H32C	0.9800	C66—H66C	0.9800
C33—C34	1.522 (3)	C67—C68	1.519 (3)
C33—H33A	0.9900	C67—H67A	0.9900
C33—H33B	0.9900	C67—H67B	0.9900
C34—H34A	0.9800	C68—H68A	0.9800
C34—H34B	0.9800	C68—H68B	0.9800
C34—H34C	0.9800	C68—H68C	0.9800
			
C2—C1—C13	100.90 (17)	C36—C35—C47	101.28 (17)
C2—C1—C29	111.98 (19)	C36—C35—C61	112.77 (19)
C13—C1—C29	111.30 (18)	C47—C35—C61	112.22 (19)
C2—C1—C27	111.39 (19)	C36—C35—C63	110.72 (19)
C13—C1—C27	110.58 (18)	C47—C35—C63	110.34 (18)
C29—C1—C27	110.38 (19)	C61—C35—C63	109.32 (19)
C3—C2—C7	120.2 (2)	C37—C36—C41	120.1 (2)
C3—C2—C1	128.5 (2)	C37—C36—C35	128.9 (2)
C7—C2—C1	111.33 (19)	C41—C36—C35	110.94 (19)
C2—C3—C4	118.8 (2)	C36—C37—C38	119.4 (2)
C2—C3—H3	120.6	C36—C37—H37	120.3
C4—C3—H3	120.6	C38—C37—H37	120.3
C5—C4—C3	120.8 (2)	C39—C38—C37	120.3 (2)
C5—C4—H4	119.6	C39—C38—H38	119.8
C3—C4—H4	119.6	C37—C38—H38	119.8
C6—C5—C4	120.5 (2)	C40—C39—C38	120.6 (2)
C6—C5—H5	119.7	C40—C39—H39	119.7
C4—C5—H5	119.7	C38—C39—H39	119.7
C5—C6—C7	118.8 (2)	C39—C40—C41	119.2 (2)
C5—C6—H6	120.6	C39—C40—H40	120.4
C7—C6—H6	120.6	C41—C40—H40	120.4
C6—C7—C2	121.0 (2)	C40—C41—C36	120.3 (2)
C6—C7—C8	130.8 (2)	C40—C41—C42	131.2 (2)
C2—C7—C8	108.17 (18)	C36—C41—C42	108.52 (19)
C9—C8—C13	119.96 (19)	C43—C42—C47	120.0 (2)
C9—C8—C7	131.27 (19)	C43—C42—C41	131.43 (19)
C13—C8—C7	108.77 (18)	C47—C42—C41	108.61 (18)
C10—C9—C8	119.05 (19)	C44—C43—C42	119.20 (19)
C10—C9—H9	120.5	C44—C43—H43	120.4
C8—C9—H9	120.5	C42—C43—H43	120.4
C9—C10—C11	121.96 (19)	C43—C44—C45	121.89 (19)
C9—C10—H10	119.0	C43—C44—H44	119.1
C11—C10—H10	119.0	C45—C44—H44	119.1
C12—C11—C10	118.24 (19)	C44—C45—C46	118.03 (19)
C12—C11—C14	121.58 (18)	C44—C45—C48	120.27 (18)
C10—C11—C14	120.14 (18)	C46—C45—C48	121.70 (18)
C13—C12—C11	119.97 (19)	C47—C46—C45	120.52 (19)
C13—C12—H12	120.0	C47—C46—H46	119.7
C11—C12—H12	120.0	C45—C46—H46	119.7
C12—C13—C8	120.82 (19)	C46—C47—C42	120.34 (19)
C12—C13—C1	128.36 (19)	C46—C47—C35	129.15 (19)
C8—C13—C1	110.82 (18)	C42—C47—C35	110.50 (18)
C26—C14—C15	117.96 (19)	C60—C48—C49	118.57 (19)
C26—C14—C11	121.23 (19)	C60—C48—C45	119.50 (18)
C15—C14—C11	120.77 (18)	C49—C48—C45	121.93 (18)
C16—C15—C14	120.35 (19)	C50—C49—C48	119.98 (18)
C16—C15—H15	119.8	C50—C49—H49	120.0
C14—C15—H15	119.8	C48—C49—H49	120.0
C15—C16—C24	120.85 (19)	C49—C50—C58	120.47 (19)
C15—C16—C17	128.28 (19)	C49—C50—C51	129.25 (18)
C24—C16—C17	110.83 (18)	C58—C50—C51	110.26 (18)
C16—C17—C18	101.15 (17)	C50—C51—C52	101.41 (17)
C16—C17—C33	113.38 (18)	C50—C51—C67	112.91 (18)
C18—C17—C33	113.56 (18)	C52—C51—C67	113.67 (19)
C16—C17—C31	109.23 (18)	C50—C51—C65	110.23 (18)
C18—C17—C31	110.45 (19)	C52—C51—C65	109.73 (18)
C33—C17—C31	108.86 (18)	C67—C51—C65	108.73 (18)
C19—C18—C23	119.8 (2)	C53—C52—C57	120.2 (2)
C19—C18—C17	129.2 (2)	C53—C52—C51	129.0 (2)
C23—C18—C17	110.95 (19)	C57—C52—C51	110.72 (19)
C20—C19—C18	118.9 (2)	C52—C53—C54	119.1 (2)
C20—C19—H19	120.5	C52—C53—H53	120.4
C18—C19—H19	120.5	C54—C53—H53	120.4
C21—C20—C19	120.9 (2)	C55—C54—C53	120.6 (2)
C21—C20—H20	119.6	C55—C54—H54	119.7
C19—C20—H20	119.6	C53—C54—H54	119.7
C20—C21—C22	120.6 (2)	C56—C55—C54	120.6 (2)
C20—C21—H21	119.7	C56—C55—H55	119.7
C22—C21—H21	119.7	C54—C55—H55	119.7
C23—C22—C21	118.5 (2)	C55—C56—C57	118.8 (2)
C23—C22—H22	120.7	C55—C56—H56	120.6
C21—C22—H22	120.7	C57—C56—H56	120.6
C22—C23—C18	121.2 (2)	C56—C57—C52	120.6 (2)
C22—C23—C24	130.6 (2)	C56—C57—C58	130.9 (2)
C18—C23—C24	108.22 (19)	C52—C57—C58	108.44 (18)
C25—C24—C16	119.91 (19)	C59—C58—C50	120.14 (19)
C25—C24—C23	131.6 (2)	C59—C58—C57	131.10 (19)
C16—C24—C23	108.49 (18)	C50—C58—C57	108.74 (18)
C24—C25—C26	119.1 (2)	C60—C59—C58	119.15 (19)
C24—C25—H25	120.5	C60—C59—H59	120.4
C26—C25—H25	120.5	C58—C59—H59	120.4
C25—C26—C14	121.8 (2)	C59—C60—C48	121.68 (19)
C25—C26—H26	119.1	C59—C60—H60	119.2
C14—C26—H26	119.1	C48—C60—H60	119.2
C28—C27—C1	114.6 (2)	C62—C61—C35	115.13 (19)
C28—C27—H27A	108.6	C62—C61—H61A	108.5
C1—C27—H27A	108.6	C35—C61—H61A	108.5
C28—C27—H27B	108.6	C62—C61—H61B	108.5
C1—C27—H27B	108.6	C35—C61—H61B	108.5
H27A—C27—H27B	107.6	H61A—C61—H61B	107.5
C27—C28—H28A	109.5	C61—C62—H62A	109.5
C27—C28—H28B	109.5	C61—C62—H62B	109.5
H28A—C28—H28B	109.5	H62A—C62—H62B	109.5
C27—C28—H28C	109.5	C61—C62—H62C	109.5
H28A—C28—H28C	109.5	H62A—C62—H62C	109.5
H28B—C28—H28C	109.5	H62B—C62—H62C	109.5
C30—C29—C1	114.36 (18)	C64—C63—C35	115.0 (2)
C30—C29—H29A	108.7	C64—C63—H63A	108.5
C1—C29—H29A	108.7	C35—C63—H63A	108.5
C30—C29—H29B	108.7	C64—C63—H63B	108.5
C1—C29—H29B	108.7	C35—C63—H63B	108.5
H29A—C29—H29B	107.6	H63A—C63—H63B	107.5
C29—C30—H30A	109.5	C63—C64—H64A	109.5
C29—C30—H30B	109.5	C63—C64—H64B	109.5
H30A—C30—H30B	109.5	H64A—C64—H64B	109.5
C29—C30—H30C	109.5	C63—C64—H64C	109.5
H30A—C30—H30C	109.5	H64A—C64—H64C	109.5
H30B—C30—H30C	109.5	H64B—C64—H64C	109.5
C32—C31—C17	115.2 (2)	C66—C65—C51	115.11 (19)
C32—C31—H31A	108.5	C66—C65—H65A	108.5
C17—C31—H31A	108.5	C51—C65—H65A	108.5
C32—C31—H31B	108.5	C66—C65—H65B	108.5
C17—C31—H31B	108.5	C51—C65—H65B	108.5
H31A—C31—H31B	107.5	H65A—C65—H65B	107.5
C31—C32—H32A	109.5	C65—C66—H66A	109.5
C31—C32—H32B	109.5	C65—C66—H66B	109.5
H32A—C32—H32B	109.5	H66A—C66—H66B	109.5
C31—C32—H32C	109.5	C65—C66—H66C	109.5
H32A—C32—H32C	109.5	H66A—C66—H66C	109.5
H32B—C32—H32C	109.5	H66B—C66—H66C	109.5
C34—C33—C17	115.01 (18)	C68—C67—C51	115.13 (19)
C34—C33—H33A	108.5	C68—C67—H67A	108.5
C17—C33—H33A	108.5	C51—C67—H67A	108.5
C34—C33—H33B	108.5	C68—C67—H67B	108.5
C17—C33—H33B	108.5	C51—C67—H67B	108.5
H33A—C33—H33B	107.5	H67A—C67—H67B	107.5
C33—C34—H34A	109.5	C67—C68—H68A	109.5
C33—C34—H34B	109.5	C67—C68—H68B	109.5
H34A—C34—H34B	109.5	H68A—C68—H68B	109.5
C33—C34—H34C	109.5	C67—C68—H68C	109.5
H34A—C34—H34C	109.5	H68A—C68—H68C	109.5
H34B—C34—H34C	109.5	H68B—C68—H68C	109.5
			
C13—C1—C2—C3	−178.7 (2)	C47—C35—C36—C37	178.8 (2)
C29—C1—C2—C3	−60.2 (3)	C61—C35—C36—C37	58.6 (3)
C27—C1—C2—C3	63.9 (3)	C63—C35—C36—C37	−64.2 (3)
C13—C1—C2—C7	0.4 (2)	C47—C35—C36—C41	−2.5 (2)
C29—C1—C2—C7	118.9 (2)	C61—C35—C36—C41	−122.7 (2)
C27—C1—C2—C7	−116.9 (2)	C63—C35—C36—C41	114.5 (2)
C7—C2—C3—C4	−0.9 (4)	C41—C36—C37—C38	1.5 (3)
C1—C2—C3—C4	178.1 (2)	C35—C36—C37—C38	−179.9 (2)
C2—C3—C4—C5	0.1 (4)	C36—C37—C38—C39	−0.9 (4)
C3—C4—C5—C6	0.5 (4)	C37—C38—C39—C40	0.1 (4)
C4—C5—C6—C7	−0.3 (3)	C38—C39—C40—C41	0.0 (4)
C5—C6—C7—C2	−0.5 (3)	C39—C40—C41—C36	0.6 (3)
C5—C6—C7—C8	−178.3 (2)	C39—C40—C41—C42	179.8 (2)
C3—C2—C7—C6	1.1 (3)	C37—C36—C41—C40	−1.4 (3)
C1—C2—C7—C6	−178.08 (19)	C35—C36—C41—C40	179.8 (2)
C3—C2—C7—C8	179.3 (2)	C37—C36—C41—C42	179.2 (2)
C1—C2—C7—C8	0.1 (2)	C35—C36—C41—C42	0.4 (2)
C6—C7—C8—C9	−2.0 (4)	C40—C41—C42—C43	2.9 (4)
C2—C7—C8—C9	−180.0 (2)	C36—C41—C42—C43	−177.9 (2)
C6—C7—C8—C13	177.2 (2)	C40—C41—C42—C47	−177.1 (2)
C2—C7—C8—C13	−0.7 (2)	C36—C41—C42—C47	2.2 (2)
C13—C8—C9—C10	−0.6 (3)	C47—C42—C43—C44	1.1 (3)
C7—C8—C9—C10	178.6 (2)	C41—C42—C43—C44	−178.8 (2)
C8—C9—C10—C11	−0.1 (3)	C42—C43—C44—C45	0.9 (3)
C9—C10—C11—C12	0.6 (3)	C43—C44—C45—C46	−1.5 (3)
C9—C10—C11—C14	−177.13 (19)	C43—C44—C45—C48	179.1 (2)
C10—C11—C12—C13	−0.5 (3)	C44—C45—C46—C47	−0.1 (3)
C14—C11—C12—C13	177.22 (18)	C48—C45—C46—C47	179.35 (19)
C11—C12—C13—C8	−0.1 (3)	C45—C46—C47—C42	2.1 (3)
C11—C12—C13—C1	−179.8 (2)	C45—C46—C47—C35	−176.5 (2)
C9—C8—C13—C12	0.7 (3)	C43—C42—C47—C46	−2.7 (3)
C7—C8—C13—C12	−178.66 (19)	C41—C42—C47—C46	177.29 (19)
C9—C8—C13—C1	−179.60 (19)	C43—C42—C47—C35	176.16 (19)
C7—C8—C13—C1	1.0 (2)	C41—C42—C47—C35	−3.9 (2)
C2—C1—C13—C12	178.8 (2)	C36—C35—C47—C46	−177.4 (2)
C29—C1—C13—C12	59.8 (3)	C61—C35—C47—C46	−56.9 (3)
C27—C1—C13—C12	−63.3 (3)	C63—C35—C47—C46	65.3 (3)
C2—C1—C13—C8	−0.9 (2)	C36—C35—C47—C42	3.9 (2)
C29—C1—C13—C8	−119.9 (2)	C61—C35—C47—C42	124.4 (2)
C27—C1—C13—C8	117.1 (2)	C63—C35—C47—C42	−113.4 (2)
C12—C11—C14—C26	−28.5 (3)	C44—C45—C48—C60	40.5 (3)
C10—C11—C14—C26	149.19 (19)	C46—C45—C48—C60	−138.9 (2)
C12—C11—C14—C15	153.81 (19)	C44—C45—C48—C49	−139.9 (2)
C10—C11—C14—C15	−28.5 (3)	C46—C45—C48—C49	40.7 (3)
C26—C14—C15—C16	−1.6 (3)	C60—C48—C49—C50	1.1 (3)
C11—C14—C15—C16	176.17 (18)	C45—C48—C49—C50	−178.48 (19)
C14—C15—C16—C24	0.0 (3)	C48—C49—C50—C58	−0.1 (3)
C14—C15—C16—C17	177.69 (19)	C48—C49—C50—C51	−178.5 (2)
C15—C16—C17—C18	176.2 (2)	C49—C50—C51—C52	−175.5 (2)
C24—C16—C17—C18	−5.9 (2)	C58—C50—C51—C52	6.0 (2)
C15—C16—C17—C33	54.3 (3)	C49—C50—C51—C67	−53.5 (3)
C24—C16—C17—C33	−127.80 (19)	C58—C50—C51—C67	128.04 (19)
C15—C16—C17—C31	−67.3 (3)	C49—C50—C51—C65	68.4 (3)
C24—C16—C17—C31	110.6 (2)	C58—C50—C51—C65	−110.1 (2)
C16—C17—C18—C19	−175.7 (2)	C50—C51—C52—C53	176.2 (2)
C33—C17—C18—C19	−53.9 (3)	C67—C51—C52—C53	54.7 (3)
C31—C17—C18—C19	68.7 (3)	C65—C51—C52—C53	−67.2 (3)
C16—C17—C18—C23	5.7 (2)	C50—C51—C52—C57	−6.3 (2)
C33—C17—C18—C23	127.6 (2)	C67—C51—C52—C57	−127.8 (2)
C31—C17—C18—C23	−109.8 (2)	C65—C51—C52—C57	110.2 (2)
C23—C18—C19—C20	0.0 (3)	C57—C52—C53—C54	0.0 (3)
C17—C18—C19—C20	−178.4 (2)	C51—C52—C53—C54	177.3 (2)
C18—C19—C20—C21	0.8 (4)	C52—C53—C54—C55	−0.9 (4)
C19—C20—C21—C22	−0.8 (4)	C53—C54—C55—C56	0.8 (4)
C20—C21—C22—C23	−0.1 (4)	C54—C55—C56—C57	0.1 (3)
C21—C22—C23—C18	0.9 (3)	C55—C56—C57—C52	−1.0 (3)
C21—C22—C23—C24	−177.2 (2)	C55—C56—C57—C58	177.4 (2)
C19—C18—C23—C22	−0.9 (3)	C53—C52—C57—C56	0.9 (3)
C17—C18—C23—C22	177.8 (2)	C51—C52—C57—C56	−176.79 (19)
C19—C18—C23—C24	177.6 (2)	C53—C52—C57—C58	−177.81 (19)
C17—C18—C23—C24	−3.7 (2)	C51—C52—C57—C58	4.5 (2)
C15—C16—C24—C25	1.6 (3)	C49—C50—C58—C59	−1.0 (3)
C17—C16—C24—C25	−176.48 (18)	C51—C50—C58—C59	177.66 (18)
C15—C16—C24—C23	−177.86 (18)	C49—C50—C58—C57	177.56 (18)
C17—C16—C24—C23	4.1 (2)	C51—C50—C58—C57	−3.8 (2)
C22—C23—C24—C25	−1.3 (4)	C56—C57—C58—C59	−0.6 (4)
C18—C23—C24—C25	−179.6 (2)	C52—C57—C58—C59	177.9 (2)
C22—C23—C24—C16	178.1 (2)	C56—C57—C58—C50	−179.0 (2)
C18—C23—C24—C16	−0.2 (2)	C52—C57—C58—C50	−0.4 (2)
C16—C24—C25—C26	−1.5 (3)	C50—C58—C59—C60	1.1 (3)
C23—C24—C25—C26	177.9 (2)	C57—C58—C59—C60	−177.1 (2)
C24—C25—C26—C14	−0.2 (3)	C58—C59—C60—C48	−0.1 (3)
C15—C14—C26—C25	1.7 (3)	C49—C48—C60—C59	−1.0 (3)
C11—C14—C26—C25	−176.03 (19)	C45—C48—C60—C59	178.57 (19)
C2—C1—C27—C28	61.6 (3)	C36—C35—C61—C62	59.2 (3)
C13—C1—C27—C28	−49.7 (3)	C47—C35—C61—C62	−54.4 (3)
C29—C1—C27—C28	−173.3 (2)	C63—C35—C61—C62	−177.2 (2)
C2—C1—C29—C30	−57.2 (3)	C36—C35—C63—C64	−52.6 (3)
C13—C1—C29—C30	54.9 (3)	C47—C35—C63—C64	58.7 (3)
C27—C1—C29—C30	178.1 (2)	C61—C35—C63—C64	−177.4 (2)
C16—C17—C31—C32	−50.0 (3)	C50—C51—C65—C66	58.4 (3)
C18—C17—C31—C32	60.4 (3)	C52—C51—C65—C66	−52.5 (3)
C33—C17—C31—C32	−174.3 (2)	C67—C51—C65—C66	−177.4 (2)
C16—C17—C33—C34	54.3 (3)	C50—C51—C67—C68	−57.4 (3)
C18—C17—C33—C34	−60.5 (3)	C52—C51—C67—C68	57.4 (3)
C31—C17—C33—C34	176.0 (2)	C65—C51—C67—C68	179.9 (2)

### Hydrogen-bond geometry (Å, º)

*Cg*3 and *Cg*4 are the centroids of the C14–C16/C24–C26 and C8–C13 rings, respectively.

**Table:**

*D*—H···*A*	*D*—H	H···*A*	*D*···*A*	*D*—H···*A*
C43—H43···*Cg*3^i^	0.95	2.64	3.49	150
C60—H60···*Cg*4^i^	0.95	3.15	3.83	130

Symmetry code: (i) −*x*, −*y*, −*z*+1.
